# Supplementary figures and images for: Key Aroma Differences in Volatile Compounds of Aged Feng-Flavored Baijiu Determined Using Sensory Descriptive Analysis and GC×GC–TOFMS
Source: Foods. 2024 May 13;13(10):1504. doi: 10.3390/foods13101504 (PMC11119998; doi:10.3390/foods13101504)

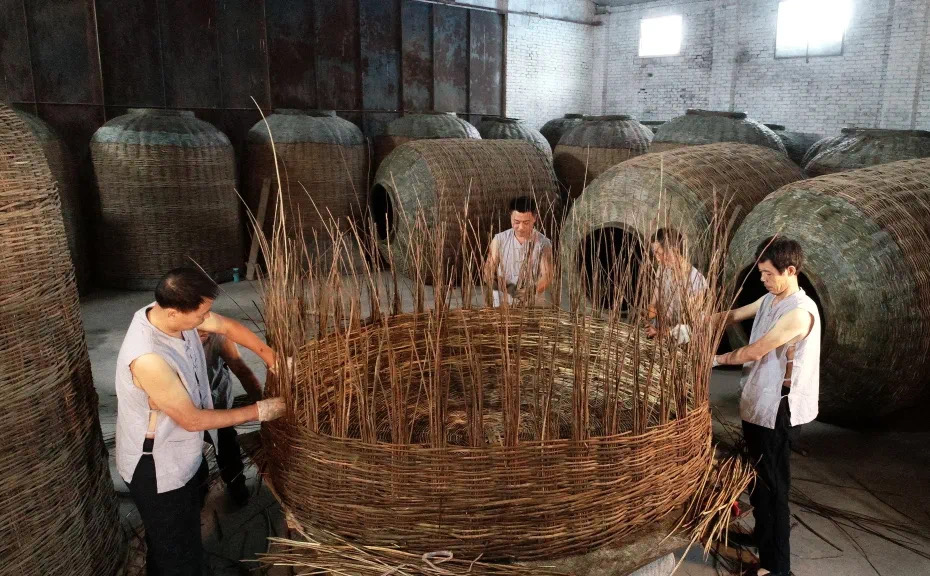

Supplement: Supplementary file 1 [file foods-13-01504-s001.zip › Figure S1 Production of Mare Nectaris.jpg]

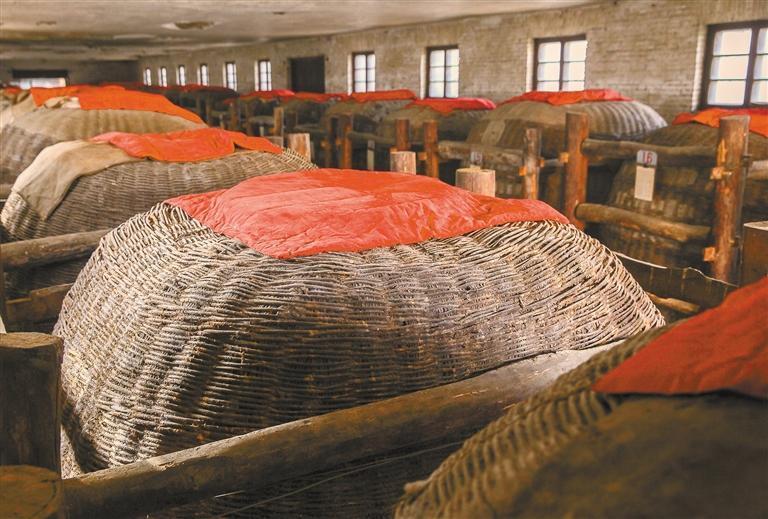

Supplement: Supplementary file 1 [file foods-13-01504-s001.zip › Figure S2 Aging of Feng-flavored baijiu in the Mare Nectaris.jpg]

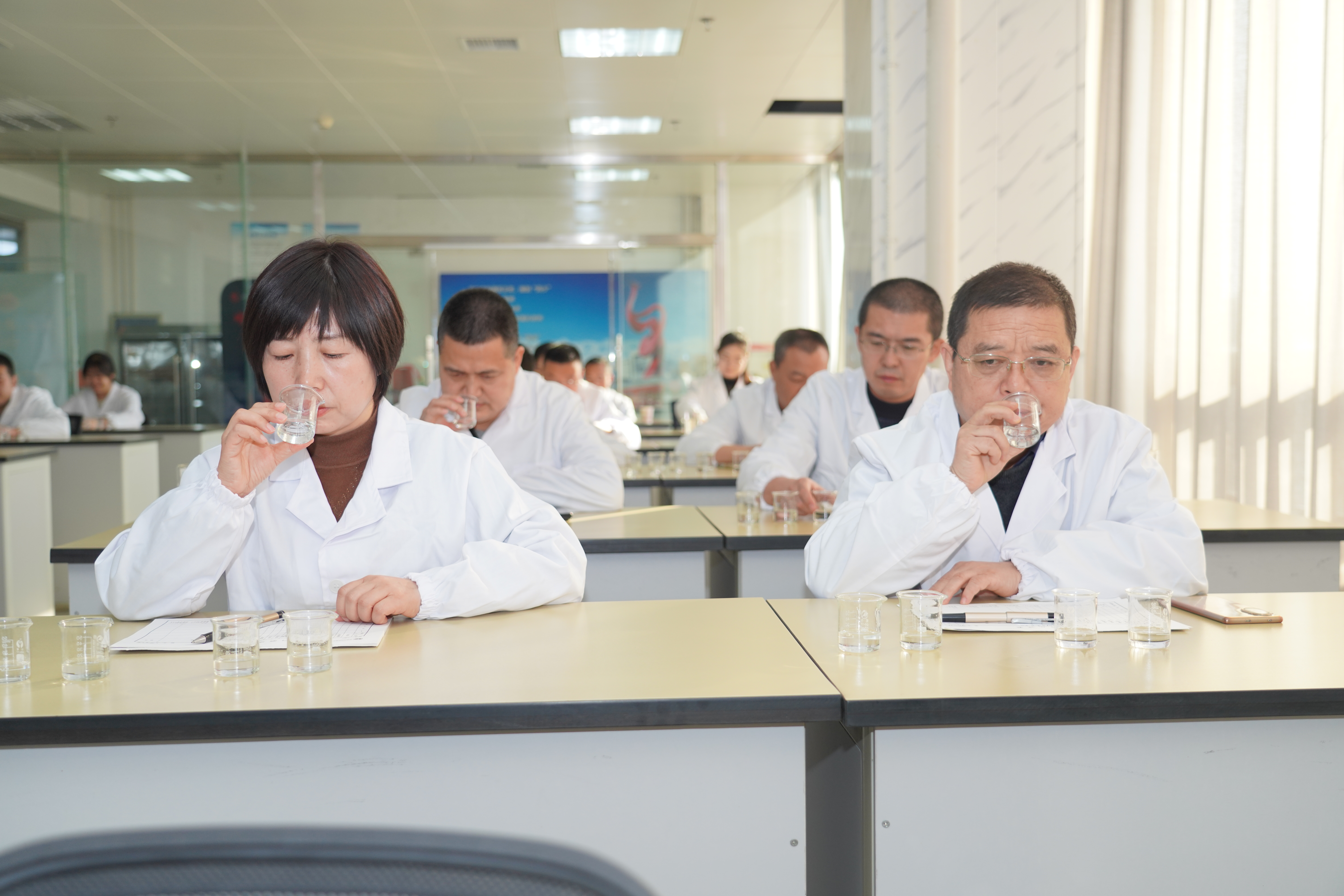

Supplement: Supplementary file 1 [file foods-13-01504-s001.zip › Figure S3 Sensory evaluation of the samples.jpg]

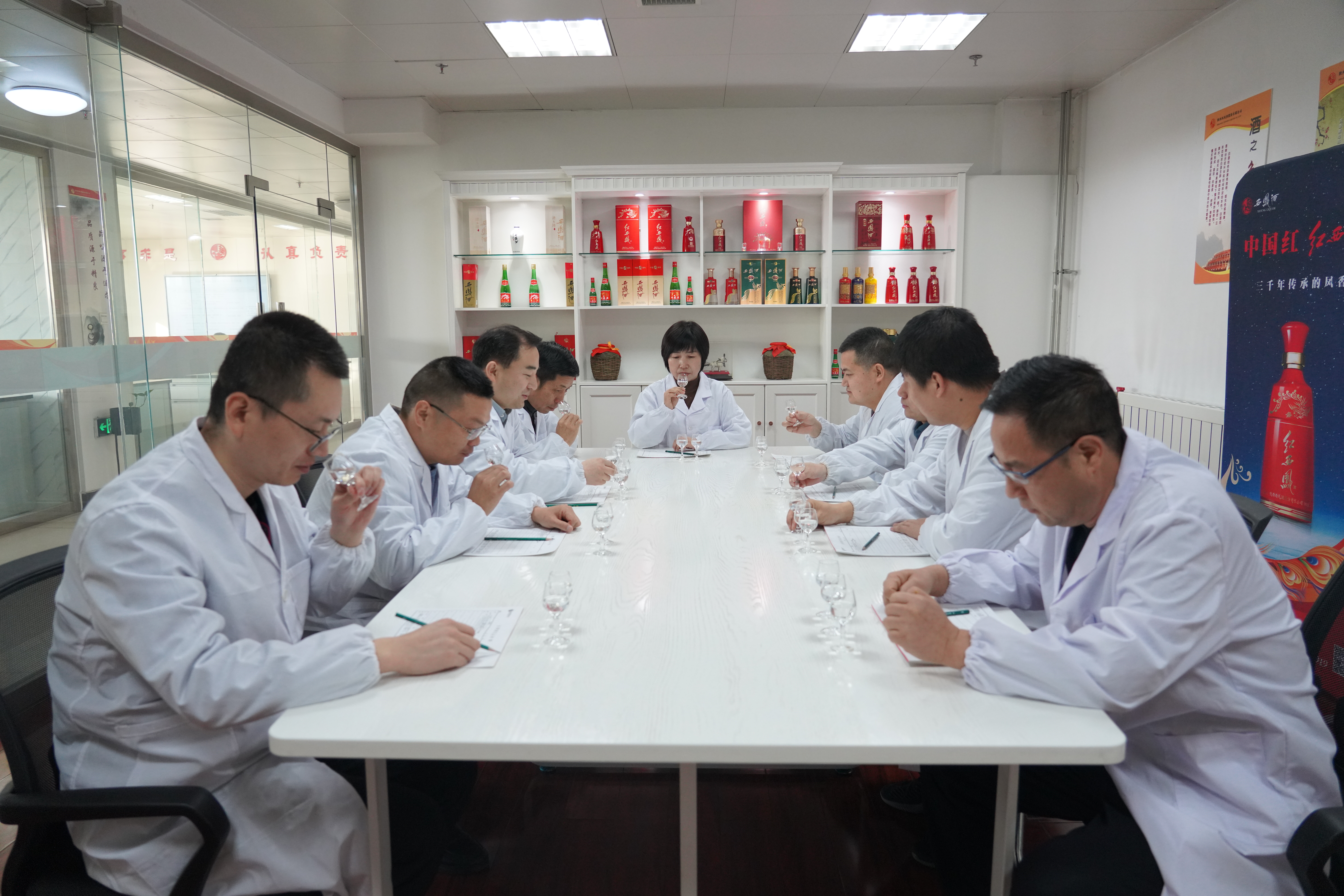

Supplement: Supplementary file 1 [file foods-13-01504-s001.zip › Figure S4 Discussion in sensory evaluation.jpg]
